# Chromone-embedded peptidomimetics and furopyrimidines as highly potent SARS-CoV-2 infection inhibitors: docking and MD simulation study

**DOI:** 10.1186/s13104-023-06508-7

**Published:** 2023-09-21

**Authors:** Zahra Shakibay Senobari, Mohsen Masoumian Hosseini, Mohammad Bagher Teimouri, Ali Hossein Rezayan, Saeed Samarghandian, Azadeh Hekmat

**Affiliations:** 1 Department of Biology, Science and Research Branch, Islamic Azad University, Tehran, Iran; 2 Department of Biochemistry, Shahid Beheshti University of Medical Sciences, Tehran, Iran; 3 Department of E-Learning in Medical Science, Tehran University of Medical Sciences, Tehran, Iran; 4 Faculty of Chemistry, Kharazmi University, Tehran, Iran; 5 Department of Life Science Engineering, Faculty of New Sciences & Technologies, University of Tehran, Tehran, 1417466191 Iran; 6 Department of Basic Medical Sciences, Neyshabur University of Medical Sciences, Neyshabur, Iran

**Keywords:** SARS-CoV-2, Molecular docking, Chromone derivatives, Infection inhibitors, Spike receptor binding domain, Main protease domain

## Abstract

**Background:**

COVID-19 is a respiratory illness caused by SARS-CoV-2. Pharmaceutical companies aim to control virus spread through effective drugs. This study investigates chromone compound derivatives’ ability to inhibit viral entry and prevent replication.

**Method:**

This study investigated the inhibitory effect of chromone-embedded peptidomimetics and furopyrimidines on 7BZ5 from Severe Acute Respiratory Syndrome CoV-2, Homo sapiens, and 6LU7 from Bat SARS-like CoV using molecular docking. The crystal structure of these proteins was obtained from the Protein Data Bank, and the inhibition site was determined using ligand binding interaction options. The 3D structure was protonated and energetically minimised using MOE software. Chromone derivatives were designed in three dimensions, and their energy was minimised using MOE 2019. The molecular drug-likeness was calculated using SwissADME, Lipinski and Benigni-Bossa’s rule, and toxicity was calculated using Toxtree v3.1.0 software. Compounds with pharmacological properties were selected for molecular docking, and interactions were assessed using MOE 2019. MD simulations of Mpro-ch-p complexes were performed to evaluate root mean square fluctuations (RMSF) and measure protein stability.

**Result:**

The pharmacokinetic tests revealed that chromone derivatives of the peptidomimetic family have acceptable pharmacokinetic activity in the human body. Some compounds, such as Ch-p1, Ch-p2, Ch-p6, Ch-p7, Ch-p12, and Ch-p13, have pronounced medicinal properties. Molecular docking revealed high affinity for binding to SARS-CoV-2 protease. Ch-p7 had the highest binding energy, likely due to its inhibitory property. A 10 ns molecular dynamics study confirmed the stability of the protein–ligand complex, resulting in minimal fluctuations in the system's backbone. The MM-GBSA analysis revealed free energies of binding of − 19.54 kcal/mol.

**Conclusions:**

The study investigated the inhibition of viral replication using chromone derivatives, finding high inhibitory effects in the peptidomimetic family compared to other studies.

## Introduction

The COVID-19 pandemic has led to severe illness due to the highly mutated wild type of SARS-CoV-2. With limited testing methods and the possibility of new variants emerging, the scientific community recommends vaccination and early treatment to prevent the disease and protect against its deadly effects [1].

The SARS-CoV-2 virus is closely related to SARS-CoV [2, 3], causing diseases like severe respiratory illness and pneumonia [4, 5]. Its structure consists of a lipid bilayer surrounding the viral envelope and anchoring structural proteins like the membrane, envelope, and spike [6]. Infection with SARS-CoV-2 depends on the interaction between the S1 protein and the receptor [7]. The receptor-binding domains of S1 bind directly to the peptidoacylase domains of angiotensin-converting enzyme 2 (ACE2) [8–10]. Detachment of the ACE2 host receptor can lead to loss of ACE2 function and systemic release of the S1/ACE2 complex [11]. Covid-19’s spike glycoprotein supports viral binding and entry [12, 13]. Binding a neutralising antibody to its glycoprotein spike prevents the virus from entering and functioning in human embryonic kidney cells [13].

The SARS-CoV-2 main protease (Mpro) is crucial for virus replication and transcription [14], cleaving pp1a and pp1ab polyproteins [2, 15]. It plays a crucial role in the life cycle of CoVs [16], and is being investigated as a drug target due to its lack of homology to human proteins [17].

Numerous drug research studies have been conducted on COVID-19 and its variants, but no specific treatment exists [18]. Antiviral drugs like Remdesivir and Paxlovid have been approved, but they were initially developed for non-hospitalized patients without chronic diseases [19–21]. This uncertainty affects COVID-19 treatment in patients with critical health conditions like cancer, diabetes, and cardiovascular problems. Investing in research and development is crucial for finding new treatments and vaccines. Natural remedies are generally better for therapy as they do not contain harmful chemicals, are less toxic, and have fewer side effects than synthetic compounds [22]. Studies have shown that chromone has several therapeutic effects [23, 24].

Chromone scaffolds are valuable in natural products, pharmaceuticals, and bioactive molecules, making them a significant part of human nutrition [25, 26]. Molecular docking studies have shown that chromones can inhibit the RBD function of the SARS-CoV-2-S protein [27], but clinical trials have not yet proven this. Chromones act as mast cell stabilisers, alleviating respiratory complications associated with CoV infections [28]. Effective drug treatments for SARS-CoV-2 are urgently needed, but testing new drugs quickly is unrealistic. One approach is to screen potential agents using computational methods to identify specific antiviral drug candidates [29]. This study investigated the pharmacokinetics of chromone derivatives from the peptidomimetics and furopyrimidines, followed by molecular docking and molecular dynamics analysis to investigate the inhibitory activity of selected medicinal compounds against the spike receptor and Mpro of SARS CoV-2.

## Material and method

Molecular processing was performed on Windows 10 PCs with Intel Core i7 7700 K processors and NVidia GeForce GTX 1080 graphics cards. The Molecular Operating Environment (MOE) 2019 was used for ligand design and molecular docking [30]. SwissADME and Toxtree v3.1.0 software calculated pharmacokinetics, bioavailability, and toxicity [31, 32]. Molecular dynamics (MD) simulations of protein–ligand complexes were performed using CABS-flex V 2.0 (http://biocomp.chem.uw.edu.pl/CABSflex2) and the iMODS server (http://imods.chaco nlab.org).

### Molecular design of chromone derivative compounds

This study investigated two families of compounds containing peptidomimetics and furopyrimidines embedded with chromones. Table 1 shows the structure of the compounds from the peptidomimetic and furopyrimidine families selected for this study. The chromone derivatives, which differ in the location of the benzene groups, were simulated in two dimensions (2D) and three dimensions (3D). Chromone derivatives were designed in 3D using MOE software and prepared with standard parameters. The software MOE minimized energy by adjusting the force field setting MMFF94 and obtaining the gradient setting RMS of 0.001 kcal/mol.Å. A. The *mdb database was then created for each compound.

**Table 1:**
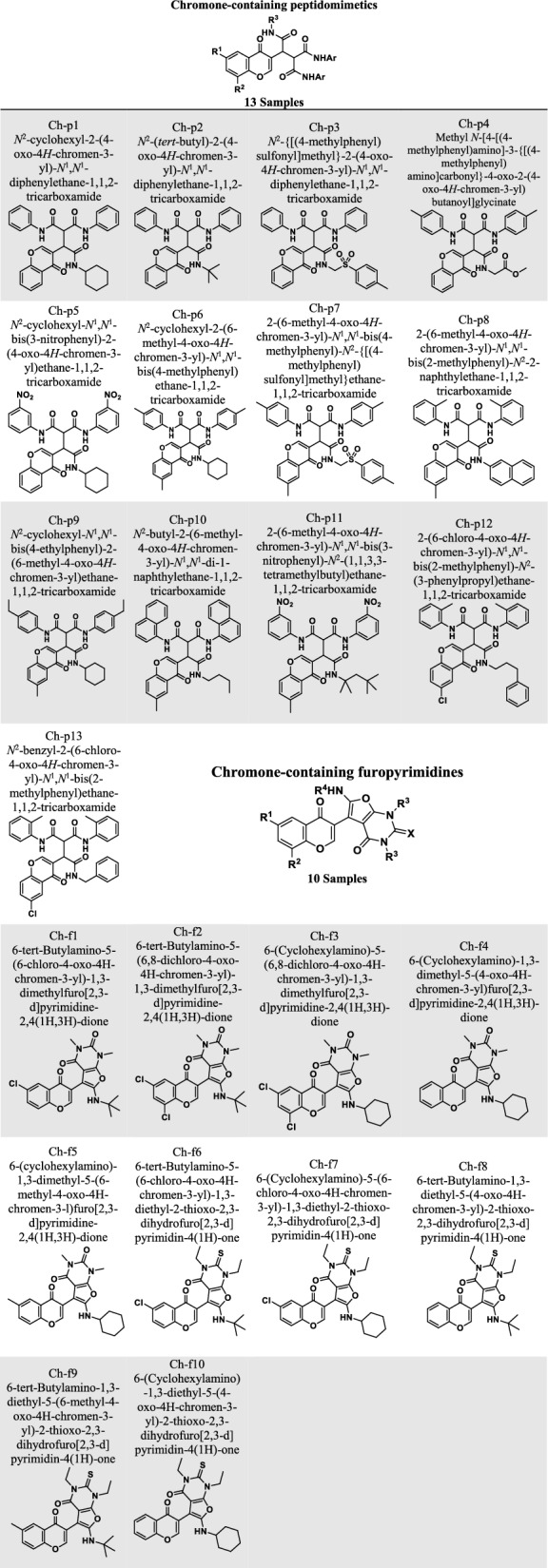
The structure of chromone derivatives from the family of peptidomimetics and furopyrimidines

The online software SwissADME to determine intrinsic properties and Lipinski's rule or ADME (Absorption, Distribution, Metabolism, and Excretion) were used to calculate the molecular drug-likeness of these compounds [32, 33]. According to this rule, a drug-like molecule has a logP ≤ 5, a molar mass < 500 g/mol, hydrogen bond acceptors ≤ 10, and hydrogen bond donors ≤ 5. A high drug-likeness similarity value means that the molecule contains fragments commonly found in commercially available drugs. Drug candidates that violate more than one of these rules are unlikely to be effective [34]. The Lipinski rule excluded the undesirable chromone derivative compounds, and the remaining compounds were prepared for the subsequent steps.

### Structure preparation of protein

As shown in Table 2, two structures of SARS-CoV-2, the spike receptor binding domain and the crystal structure of Mpro were obtained in PDB format from the Protein Data Bank (http://www.rcsb.org). The structure PDB ID: 7BZ5 belonged to the spike receptor binding domain bound with a neutralizing antibody [13]. The structure of 6LU7 belonged to the crystal structure of Mpro inhibited by the N3 inhibitor [14]. During structure preparation, unwanted molecules such as water molecules and NAG (2-acetamido-2-deoxy-beta-d-glucopyranose) in 7BZ5 and peptide-like (N3) in 6LU7 were removed via MOE. In addition, antibodies bound to 7BZ5 and the N3 inhibitor bound to 6LU7 were removed after the binding sites were determined.

**Table 2:** Crystallographic properties of proteins

Protein	PDB code	Classification	Organism	Resolution (Å)	Method	Sequence length	Chain
COVID-19 virus spike receptor-binding domain	7BZ5	Viral protein/immune system	Severe acute respiratory syndrome CoV 2, Homo sapiens	1.84	X-RAY diffraction	229	A
COVID-19 main protease	6LU7	Viral protein	Bat SARS-like CoV	2.1	X-RAY diffraction	306	A

### Preparation of proteins for the molecular docking

The QuickPrep option of MOE was used to protonate and minimize the 3D structure of proteins. Parameters like AMBER10:EHT force field, “gas phase” solvation, and an RMS gradient of 0.001 kcal/mol/A were chosen for virus spike receptor structures. Protonated 3D options were selected, and ASN/GLN/HIS-flips were allowed. The Site Finder option on MOE estimated binding sites for Mpro and viral spike receptor, and two chromone families were docked separately to these sites.

### Binding site prediction for the SARS CoV-2 spike receptor

The study used antibody-bound amino acids to predict binding sites, identifying two sites with amino acids Arg403 and Arg457. These binding sites are shown here as A and B; the amino acids are shown in Table 3. These sites were used for molecular docking analysis. Dummy atoms were formed from alpha spheres. The ‘Rigid Receptor’ protocol and ‘Triangle Matcher/London dG’ and ‘Forcefield/GBVI-WSA dG’ factors were chosen for placement and refinement, respectively. Pose values of 30 and 5 were chosen for docking. The S-score was used to evaluate interactions, with inhibitors with lower S-scores interacting strongly with SARS-CoV-2 spike receptors.

**Table 3:** The possible binding sites were based on the site finder option containing amino acids Arg403 and Arg457

Site	Amino acids involved
Site A	Arg403, Glu406, Gln409, Gly416, Lys417, Ile418, Tyr453, Gln493, Ser494, Tyr495, and Tyr505
Site B	Arg454, Php456, Arg457, Lys458, Asp467, Ser469, Thr470, Glu471, Ile472, Tyr473, and Pro491

### Active site prediction for the SARS CoV-2 Mpro

**Fig. 1:**
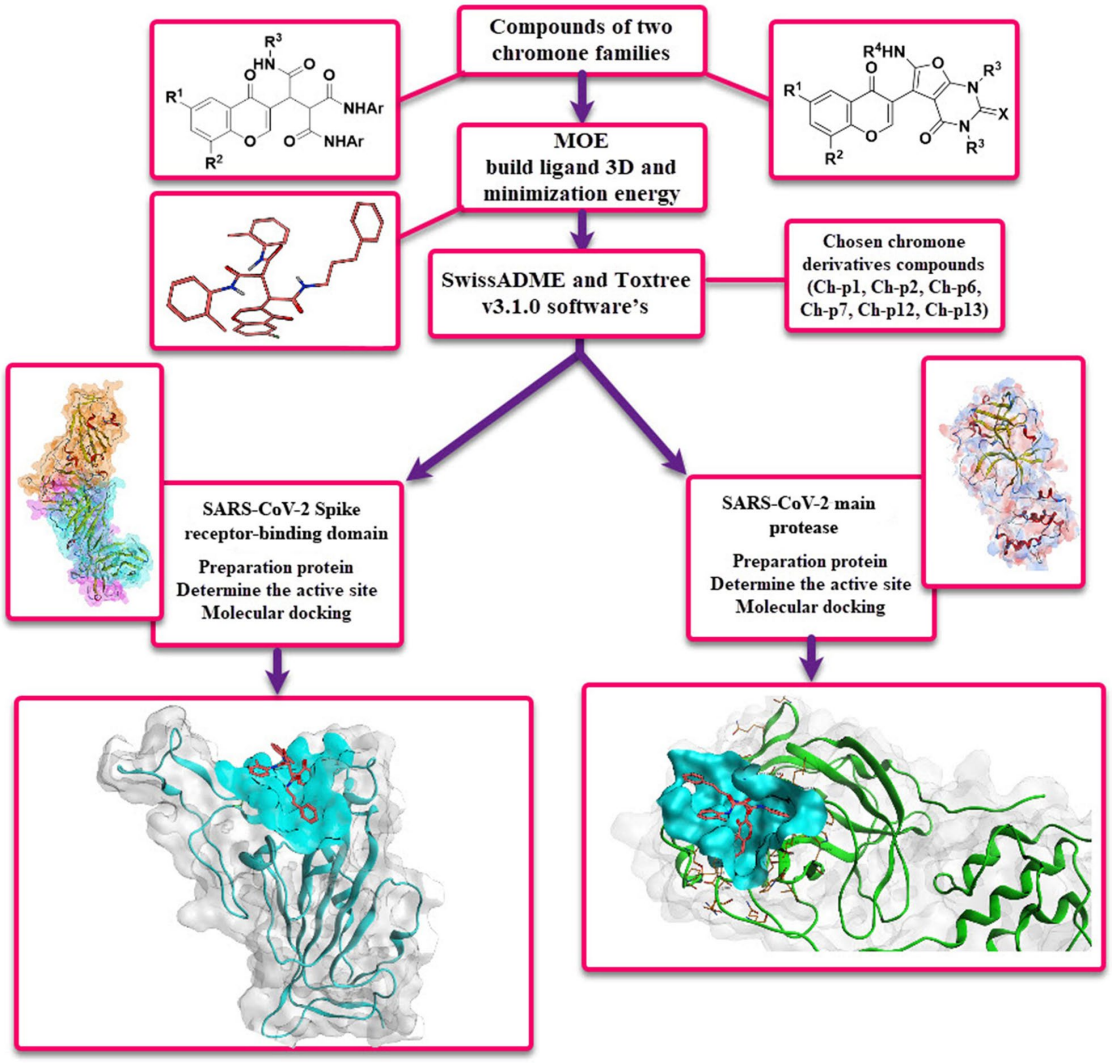
The summarized steps in selecting, preparing, and molecular docking the compounds studied

The active site of Mpro of SARS-CoV-2 also referred to as protomer A, contains residues Phe140, His172, Gly143, His164, Glu166, Met165, Gln189, and Thr190. These residues were selected and used for molecular docking. Figure 1 shows the selected compounds and the procedure for the study.

### Molecular dynamics simulation analysis

Molecular dynamics simulations (MD) were performed to assess the stability of protein–ligand complexes. The MD simulations were performed using the iMOD server to evaluate the stability of docked complexes and their physical motions. All proteins' structural flexibility (RMSF) was assessed using CABS-flex. The simulation time was set to 10 ns, while the other parameters were left at their default settings. Mean square fluctuations (RMSF) were recorded using the default options in the MD trajectory or the NMR ensemble. MD Simulations were performed using iMOD to calculate the stability and molecular motion of protein-Ch-Ps complexes docked in water. In addition to analysing the structural dynamics of the docking complexes, iMODS was used to determine the molecular motions of the docking complexes. Normal mode analysis (NMA) was performed to investigate the slow dynamic changes of the docked complexes and reveal their substantial conformational fluctuations. During the calculation, the server computes the combined motion of the large macromolecules and the NMA of the surface coordinates of the Cα atoms. In addition, iMODS estimates the atomic disorder (B-factor), the structural deformability and the eigenvalues. The deformability value measures how well the protein can adapt to different conformations, while the B-factor measures the mobility of the protein backbone. By combining these two metrics, we can determine the mobility profiles of the proteins, which is important for understanding how proteins behave in different environments. We used docked PDB files in the input files. The files were uploaded to the iMODS server with all parameters set to default values.

### MM-GBSA and MM-PBSA analysis

To determine the free energies of binding of the protein and ligand complexes, an MM-GBSA (Molecular Mechanics, Generalised Born Model, Solvent Accessibility) analysis was performed. To calculate the optimal binding energy, the Prime module of the Schrödinger software was used to calculate the docking scores of the selected complexes with the lowest docking scores. In this analysis, the VSGB 2.0 model was used, which contains OPLS-AA force fields coded with implicit solvent models and modifications related to π–π interactions, hydrophobic interactions and hydrogen bond-self contact interactions. The MM /PBSA analysis was performed using the MOE PBSA solvation energy calculator plugin. The solvent was NaCl, and the concentration and temperature were determined with 0.1 M and 300 K, respectively. The results of MM-GBSA and MM-PBSA are shown in Table 8.

## Result

### Drug-like properties of chromone derivatives

The twenty-two compounds from two chromone families were tested for toxicity and pharmacological properties to ensure that these ligands function as drugs and can be taken orally. As shown in Table 4, all compounds except Ch-p6 and Ch-p11 did not violate more than one of Lipinski’s RO5 rules in this phase. The bioavailability values of all compounds were the same, as shown in Table 5. However, one of them (Ch-p11) had a value of 0.17, indicating that it has worse bioavailability than all other compounds. Nevertheless, gastrointestinal absorption of all ligands of the peptidomimetic family was low, except for Ch-p2. Moreover, this parameter was high for most of the furopyrimidine family, except for compounds Ch-f6, Ch-f7, Ch-f9, and Ch-f10. According to these data, the body can only absorb compounds Ch-p2, Ch-f1, Ch-f2, Ch-f3, Ch-f4, Ch-f5, and Ch-f8 from these two families.

**Table 4:** Lipinski’s rule of five for ADME analysis of pharmacokinetic properties of chromone derivatives from the family of peptidomimetics and furopyrimidines

No	Name	Molecular weight (g/mol)	Lipophilicity (MLog *P*)	Hydrogen bond donors	Hydrogen bond acceptors	Lipinski’s rule follows
		Less than 500 Dalton	Less than 5	Less than 5	Less than 10	Less than 2 violations
1	Ch-p1	537.61	2.28	3	5	Yes
2	Ch-p2	511.57	1.90	3	5	Yes
3	Ch-p3	609.65	1.67	3	7	Yes
4	Ch-p4	555.58	1.29	3	7	Yes
5	Ch-p5	627.60	0.70	3	9	Yes
6	Ch-p6	579.69	2.83	3	5	NO
7	Ch-p7	665.75	2.39	3	7	Yes
8	Ch-p8	609.67	3.27	3	5	Yes
9	Ch-p9	607.74	3.19	3	5	Yes
10	Ch-p10	625.71	3.21	3	5	Yes
11	Ch-p11	671.70	1.25	3	9	NO
12	Ch-p12	636.14	3.43	3	5	Yes
13	Ch-p13	608.08	3.08	3	5	Yes
14	Ch-f1	429.85	2.57	1	5	Yes
15	Ch-f2	464.30	3.06	1	5	Yes
16	Ch-f3	490.34	3.48	1	5	Yes
17	Ch-f4	421.45	2.52	1	5	Yes
18	Ch-f5	435.47	2.73	1	5	Yes
19	Ch-f6	473.97	2.98	1	4	Yes
20	Ch-f7	500.01	3.39	1	4	Yes
21	Ch-f8	439.53	2.50	1	4	Yes
22	Ch-f9	453.55	2.71	1	4	Yes
23	Ch-f10	465.56	2.92	1	4	Yes

**Table 5:** The pharmacokinetic properties of chromone derivatives from the family of peptidomimetics and furopyrimidines

No	Name	GI absorption	Bioavailability Score	CYP inhibitor	Toxicity	PAINS
1	Ch-p1	low	0.55	CYP2C19, CYP2C9, CYP2D6, CYP3A4	No	0
2	Ch-p2	High	0.55	CYP2C19, CYP2C9, CYP2D6, CYP3A4	No	0
3	Ch-p3	Low	0.55	CYP1A2, CYP2C9, CYP3A4	No	0
4	Ch-p4	Low	0.55	CYP2C9, CYP2D6, CYP3A4	No	0
5	Ch-p5	Low	0.17	CYP2C9, CYP3A4	Yes	0
6	Ch-p6	Low	0.55	CYP1A2, CYP2C19, CYP2C9, CYP2D6, CYP3A4	No	0
7	Ch-p7	Low	0.55	CYP1A2, CYP2C9, CYP3A4	No	0
8	Ch-p8	Low	0.55	CYP1A2, CYP2C9, CYP3A4	No	0
9	Ch-p9	Low	0.55	CYP1A2, CYP2C19, CYP2C9, CYP2D6, CYP3A4	No	0
10	Ch-p10	Low	0.55	CYP1A2, CYP2C19, CYP3A4	No	0
11	Ch-p11	Low	0.17	CYP2C9, CYP3A4	Yes	0
12	Ch-p12	Low	0.55	CYP1A2, CYP2C19, CYP2C9, CYP3A4	No	0
13	Ch-p13	Low	0.55	CYP1A2, CYP2C19, CYP2C9, CYP3A4	No	0
14	Ch-f1	High	0.55	CYP2C9, CYP3A4	No	0
15	Ch-f2	High	0.55	CYP2C19, CYP2C9, CYP3A4	No	0
16	Ch-f3	High	0.55	CYP2C19, CYP2C9	No	0
17	Ch-f4	High	0.55	CYP2C19, CYP2C9, CYP3A4	No	0
18	Ch-f5	High	0.55	CYP2C19, CYP2C9, CYP3A4	No	0
19	Ch-f6	Low	0.55	CYP2C19, CYP2C9, CYP3A4	No	0
20	Ch-f7	Low	0.55	CYP2C19, CYP2C9, CYP3A4	No	0
21	Ch-f8	High	0.55	CYP2C19, CYP2C9, CYP3A4	No	0
22	Ch-f9	Low	0.55	CYP2C19, CYP2C9, CYP3A4	No	0
23	Ch-f10	Low	0.55	CYP2C19, CYP2C9, CYP3A4	No	0

There were no pan-assay interference compounds (PAINS) in the testing of the PAINS, indicating that none of the chromone derivatives are likely to elicit false-positive reactions in a high-throughput screen [35, 36]. Cytochromes P450 (CYPs) in the liver catalyze the conversion of compounds to other compounds, reducing their therapeutic effect [37]. As shown in Table 5, pharmaceutical calculations using SwissADME software show that compounds Ch-p1, Ch-p2, Ch-p6, Ch-p7, Ch-p12, and Ch-p13 inhibit at least four of the five CYP enzymes (CYP1A2, CYP2C19, CYP2D6, and CYP3A4).

### Interaction chromone derivatives with SARS-CoV-2

**Fig. 2:**
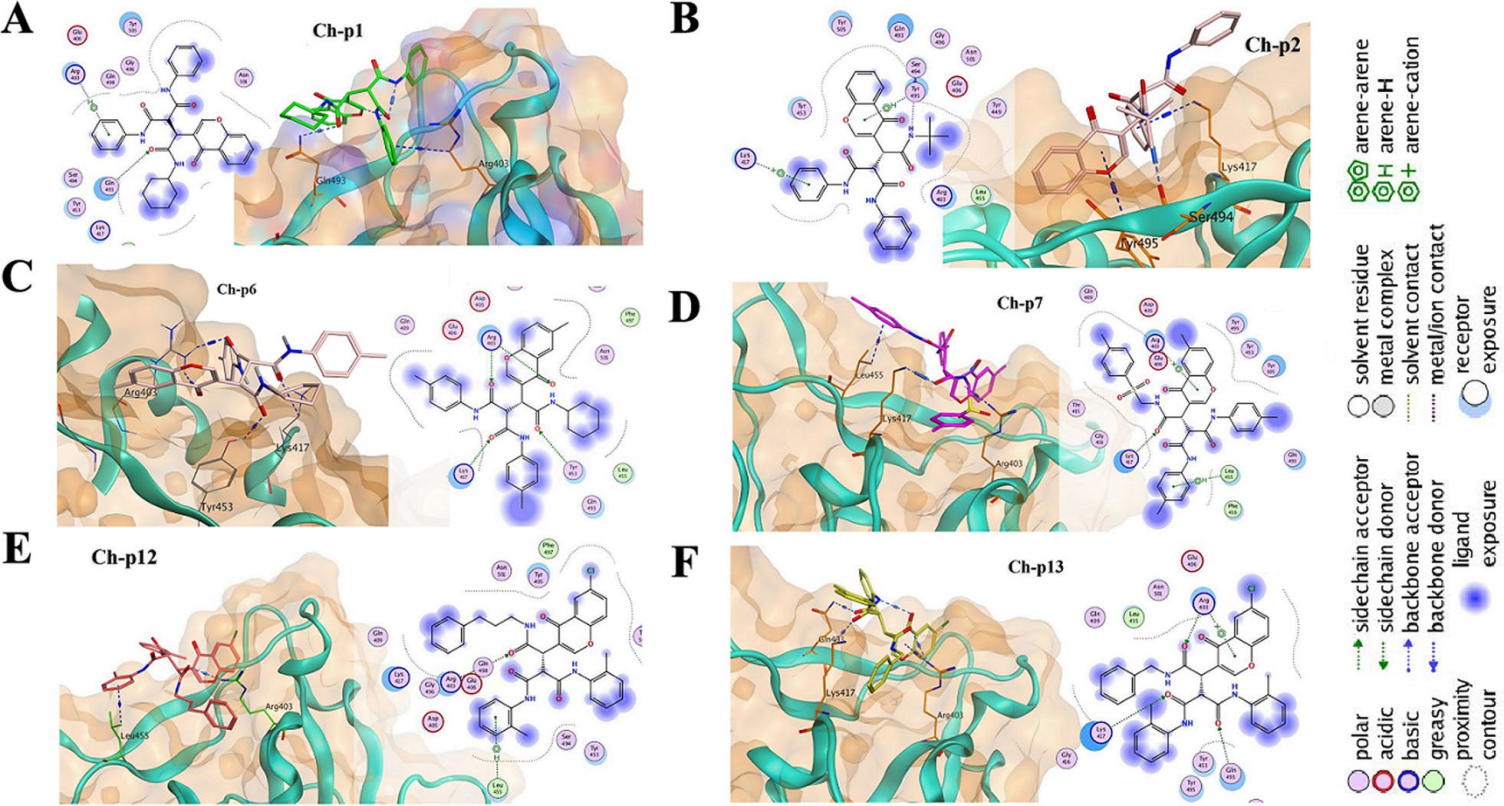
Interaction of Ch-p1, Ch-p6, Ch-p7, Ch-p12, and Ch-p13 with the SARS-CoV-2 spike receptor domain. **A** A benzene ring in Ch-p1 was bound to Arg403, and a carbonyl group was bound to Gln493. **B** Ch-p2 was bound to Lys417 via the benzene ring and to the amino acid Ser494 via the amino group. **C** Ch-p6 was bound to Lys417, Arg403, and Tyr453 in different positions via the carbonyl group. **D** Ch-p7 was bound to Leu455 via the benzene group and Lys417 via the CO group. Arg403 of SARS-CoV-2 was also bound to this compound. **E** Ch-p12 was bound to Leu455 via the benzene group and Arg403 via the CO group. **F** Ch-p13 was bound to Arg403 via two bonds and Gln493 and Lys417 via the CO group. The Arg403, Ala475, and Asn487 residues in this domain play an essential role in interacting with the human ACE2 receptor. The interaction of the ACE2 receptor with the RBD of SARS-CoV-2 is also mediated by amino acids Gln493 and Leu455. Chromone derivatives inhibit the binding of this domain to human ACE2 receptors. Therefore, the function of this protein is expected to be impaired if it does not bind to ACE2 receptors

The molecular docking result was evaluated after 300 placement poses for each ligand (Tables 6 and 7). Since most of the compounds showed greater affinity for site A than site B, these compounds likely inhibit site A more strongly. As shown in Table 6 at the spike receptor binding site, Ch-p1 at site A was bound to Arg403 via the benzene ring and Gln493 via C=O, with an S-score of − 5.7858 (Fig. 2A). Ch-p2 with an S-score of − 6.1681 was bound to amino acids Lys417, Ser494, and Tyr495 at site A (Fig. 2B). The Ch-p6 with S-score -6.0399 was bound with two bonds to Arg403 and Lys417, Tyr453 at site A (Fig. 2C). The Ch-p7 with an S-score of -6.5198 was bound in two bonds to Arg403 and Lys417 (Fig. 2D). Compound Ch-p12, like Ch-p1, Ch-p6, and Ch-p7, was bound to Arg403. However, the S score of this compound was -6.8937 (Fig. 2E). Compound Ch-p13 with an S-score of -6.0683 was bound to Arg403 with two bonds and Lys417 and Gln493 at site A (Fig. 2F).

**Table 6:** S-score with amino acids involved in the inhibition of the viral spike receptor of SARS-CoV-2

No	name	S-score	E-Conformation	Amino acid bonds
1	Ch-p1	− 5.7858	− 113.3457	Arg403, Gln493
2	Ch-p2	− 6.1681	− 169.6893	Lys417, Ser494, Tyr495
3	Ch-p6	− 6.0399	− 131.1683	Arg403, Arg403, Lys417, Tyr453
4	Ch-p7	− 6.8417	− 12.8256	Arg403, Lys417, Leu455
5	Ch-p12	− 6.8937	− 121.6345	Arg403, Leu455
6	Ch-p13	− 6.0683	− 132.5410	Arg403, Arg403, Lys417, Gln493

Amino acid binding is based on the best formation of the SARS-CoV-2-compound complex

**Table 7:** S-score with amino acids involved in inhibition of Mpro of SARS-CoV-2

No	Name	S-score	E-Conformation	Amino acid bonds
1	Ch-p1	− 8.1237	− 105.3784	Gln189, Glu166, His164, His41
2	Ch-p2	− 7.9689	− 164.9851	Glu166, Glu166, Gln189, Asn142
3	Ch-p6	− 8.4062	− 130.7555	Glu166, Cys145
4	Ch-p7	− 8.7370	− 7.3044	Gln189, Asn142, Asn142, Asn142
5	Ch-p12	− 8.7005	− 111.7998	Gln189, Gln189
6	Ch-p13	− 8.4331	− 120.3400	Gly143, Met49

Amino acid binding is based on the best formation of the SARS-CoV-2-compound complex

**Fig. 3:**
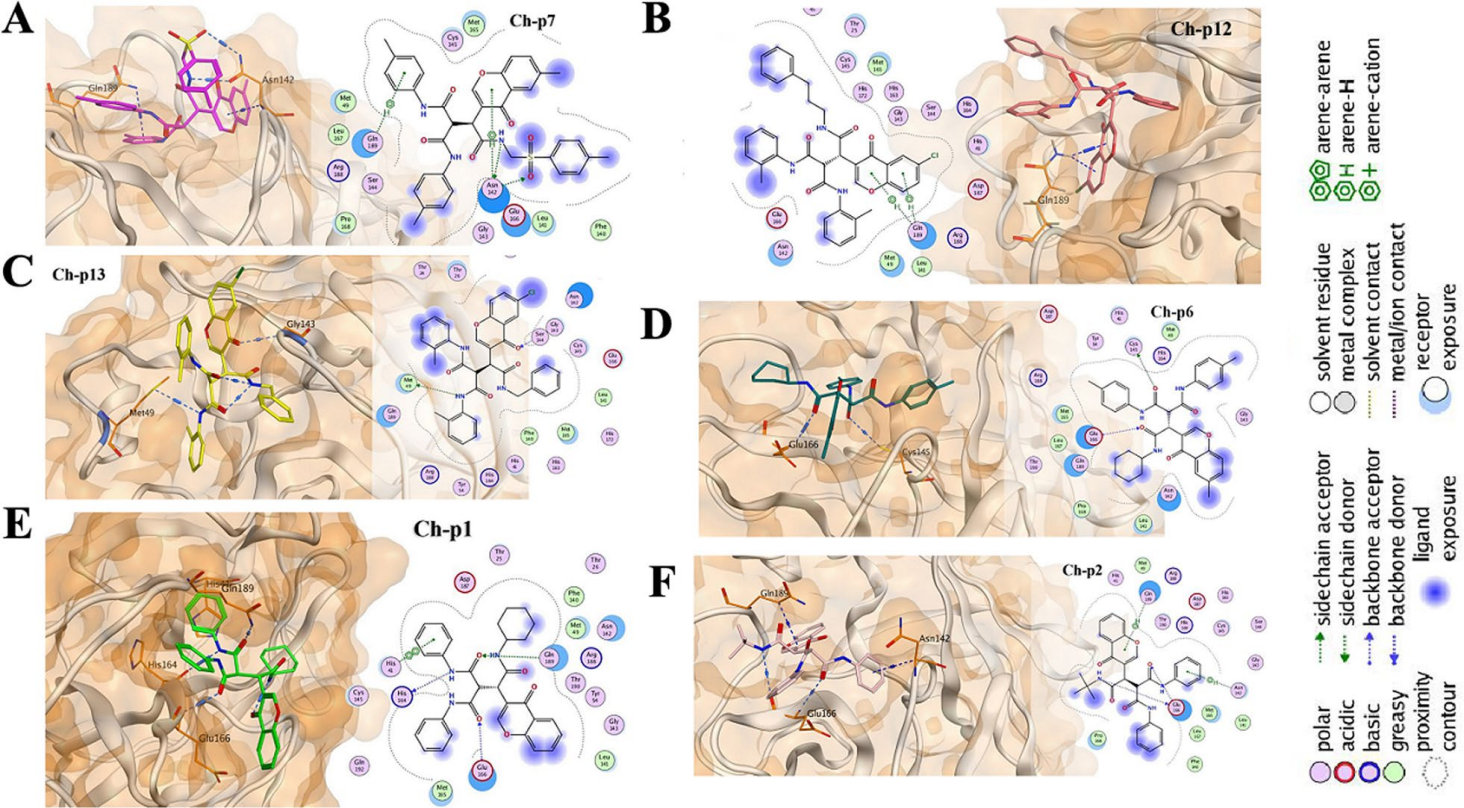
Interaction of Ch-p1, Ch-p6, Ch-p7, Ch-p12, and Ch-p13 with the Mpro of SARS-CoV-2. The Mpro of SARS-CoV-2 was inhibited by chromone derivatives binding to its primary amino acids (Phe140, His172, Gly143, His164, Glu166, Met165, Gln189, and Thr190). This site contains many essential amino acids at the active site of protomer A (Phe141, Asn142, Glu166, His163, and His 172), and inhibition of this site impairs proteolytic processing. The chromone derivatives bind to the amino acids of protomer A in the following order: Ch-p1 and Ch-p2 bind to Gln189 and Glu166, respectively. In addition, Gln189 binds to both Ch-p7 and Ch-p12. Thus, Gln189, Glu166, and His164 appear to play essential roles in the function of this protein. Among the interactions detected with the main SARS-CoV-2 protease, compound Ch-p12 had a greater tendency to inhibit protomer A, with an S-score of -8.7005

The docking results in Mpro binding site showed that most of the selected chemical compounds were bound to the protein with an S score of less than − 8. This indicated a tendency to inhibit the primary protease protomer A of SARS-CoV-2. Ch-p7 had the highest binding energy with an S-score of − 8.7370. This compound, via one bond, was bound to Gln189 and via three bonds to Asn142 (Fig. 3A). Compound Ch-p12, with an S-score of − 8.7005, was bound to Gln189 via a benzene ring with two bonds and ranked second in this group of compounds based on the inhibition S-score (Fig. 3B). The binding S-score of Ch-p13 and Ch-p6 had almost the same value (− 8.4331 and − 8.4062, respectively). However, the two compounds differed in their affinity for amino acids. Figure 3C shows compound Ch-p13 was bound to amino acids Gly143 and Met49. Compound Ch-p6 was bound to amino acid Glu166 and the -Sh group of Cys145 (Fig. 3D). Compound Ch-p1 was bound to the Mpro of SARS-CoV-2 with four amino acids Gln189, Glu166, His164, and His41 (Fig. 3E). However, the S-score for binding was − 8.1237. Regarding binding energy, Ch-p2 had the highest binding energy (S-score − 7.9689). This compound interacted with the amino acids Glu166 (with two bonds), Gln189, and Asn142 (Fig. 3F). The interactions between chromon-derived compounds and 6LU7 are listed in Table 7.

### Molecular dynamics simulation

**Fig. 4:**
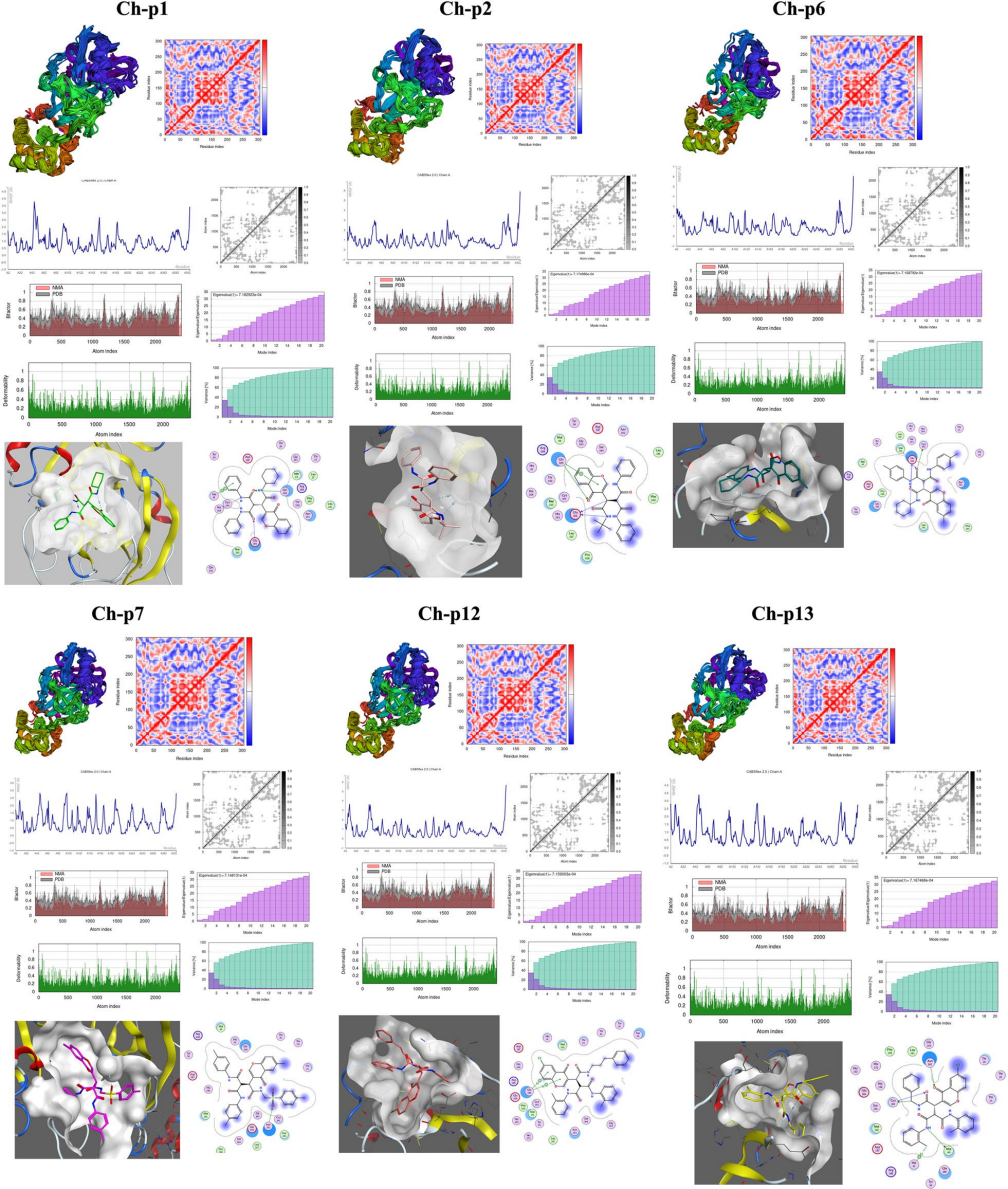
NMA results include protein domain mobility diagrams, PDB and NMA B-factor diagrams, deformation diagrams for atomic fluctuations, eigenvalue diagrams, covariance matrix diagrams, elastic network diagrams and structural flexibility (RMSF). In addition, the interaction between the ligand and the protein is shown according to MD

Using CABS-flex to evaluate the MD simulations of these six protein complexes, we found that all six proteins have multiple regions of high flexibility in their RMSF peaks. The maximum RMSF value indicates greater flexibility, while the minimum value indicates that the system was constrained throughout the simulation run. The RMSF results show that the complex Mpro-Ch-p7 is the most flexible compound, followed by Mpro- Ch-p13. The NMA of the docked complexes Mpro-Ch-p7, Mpro-Ch-p13, Mpro-Ch-p12, Mpro-Ch-p6, Mpro-Ch-p2 and Mpro-Ch-p1 is shown in Fig. 4. The eigenvalue is the amount of energy associated with a normal mode and the variance is the measure of the dispersion of the normal mode. As the eigenvalue increases, the variance decreases and vice versa. This is because the energy is concentrated in fewer modes, resulting in a decrease in variance. The complex eigenvalue and variance plots of Mpro-Ch-p are shown in Fig. 4. In the variance plot of Ch-p with Mpro, the purple shaded bars represent the individual variance, while the green shaded bars represent the cumulative variance.

### MD simulation; ligand interaction analysis

After re-analysis of the ligand interactions following the simulation of MD, the Ch-p7 compound remains bound to Asn142 and prevents Mpro from functioning under conditions similar to those in the body. The amino acid binding site of complexes Ch-p1 and Ch-p12 was not affected. However, the binding of the amino acid Asn142 was affected in the Ch-p2 compound, and the bond was broken. Furthermore, in the Ch-p13 complex, the Gly143 amino acid bond was broken, but Asn142, Cys145 and His41, amino acid bonds, were formed. A Met165 bond was also formed in the Ch-p6 complex. In addition, changes were observed in how the bands were linked in their energetic interaction (kcal/mol), as shown in Table 8.

**Table 8:**
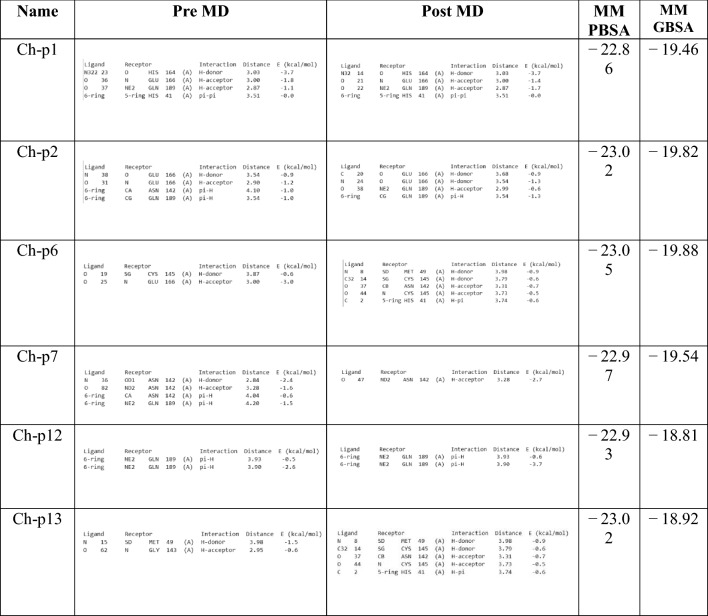
Pre- and post-MD comparison of the distance between the band interaction and the MMPBSA and MMGBSA energy

## Discussion

The peptidomimetics family, consisting of Ch-p1, Ch-p2, Ch-p6, Ch-p7, Ch-p12, and Ch-p13, has shown pharmacokinetic properties in drug-like molecules. Neutralisation of K417 is crucial for binding coronavirus RBDs to ACE2 [38], which is inhibited by binding with Ch-p2, Ch-p6, Ch-p7, and Ch-p13 compounds. The Lys31-Glu35 salt bridge in ACE2 breaks apart, forming hydrogen bonds with Gln493 of SARS-CoV-2 [39–41]. Inhibition of Gln493 with Ch-p2 and Ch-p13 may prevent the binding of SARS-CoV-2 to ACE2. The hotspot Lys31 of ACE2 is stabilised by Gln493 and Leu455 of SARS-CoV-2 [39–41]. Inhibition of these residues by Ch-p1, Ch-p12, Ch-p7, and Ch-p13 suggests that these compounds weaken this salt bridge, inhibiting virus binding.

Drug repurposing studies have mainly focused on the Mpro of CoVs [42], but viral evolution can alter the structure of the Mpro substrate-binding pocket [14]. Researchers identified potent inhibitors of the Mpro molecule using pharmacophore models and molecular docking techniques from the Marine Natural Products (MNP) library. Hydrogen bonds stabilise the ligand-enzyme complex at the active site, while hydrophobic interactions link the ligands to Met49, Met65, Leu141, and Pro168 [43]. Simulation studies have shown that residues Gln189, Cys145, His41, and His164 are essential for inhibitory ligand binding [44]. In this study, chromone derivatives from the peptidomimetic family have potent effects on the essential residues in protomer A.

Compound Ch-p7 inhibits SARS-CoV-2 spike receptor function, preventing entry into host cells. However, it is more likely to bind to the amino acid Gln189 and Asn142 in Mpro via three bonds, preventing the proteolytic function of SARS-CoV-2 in virus replication. This compound has a more substantial inhibitory effect on the Mpro of the virus, with a more impressive effect (− 8.7 vs. − 7.8) compared to molecular studies using chromone derivatives [27]. Previous studies have shown that most compounds dock to the main SARS-CoV-2 protease have an S-score of − 6 [45, 46]. However, these phytochemicals have inhibitory properties [47], but their inhibitory effect is lower than the chromone derivatives investigated in this study. Derivatives of chromone compounds have also been reported to inhibit tobacco mosaic virus, which has a single-stranded RNA genome [48]. New chromone derivatives containing dithioacetals were prepared and tested for their antiviral activity against Tomato Spotted Wilt Virus (TSWV), showing promising inhibition of TSWV [49]. Since chromone compounds have excellent antiviral properties, their derivatives are proposed to be further investigated as CoV inhibitors.

The NMA study of docked proteins revealed significant deformability in all Ch-p-complexes, with the highest peaks indicating high deformability. Low eigenvalues indicated good stability and flexibility in molecular motion. The lowest eigenvalues were found in Ch-p7, Ch-p12, Ch-p13, Ch-p6, Ch-p2, and Ch-p1, indicating easier deformability and stiffness of motion. These findings provide insight into protein conformational changes and function.

## Limitation

This study did not investigate ACE-2 inhibition because the literature review suggests that loss of ACE2 and Ang (1–7) could negatively affect the organism. SARS-CoV-2 invasion may impair ACE2/MAS signalling pathways, potentially enhancing the systemic deleterious effects of the renin–angiotensin–aldosterone system. The benefit or harm of ACE2 inhibition is unclear, but it may increase the risk of death in elderly individuals with lung injury not caused by SARS-CoV-2 invasion. Molecular docking studies and this study suggest that many inhibitory compounds have a more substantial inhibitory effect on SARS-CoV-2’s Mpro, making research to prevent virus replication more applicable.

## Conclusion

COVID-19 is caused by SARS-CoV-2, which evolves over time, impacting vaccine and therapeutic effectiveness. Chromone compounds, with low binding energy, pharmacokinetic properties, and food availability, could be developed as an effective antiviral for treating SARS-CoV infection.

## Data Availability

Upon a reasonable request, the corresponding author can provide the data set that was analyzed during this study.

## Abbreviations

SARS-CoV: Severe acute respiratory syndrome coronaviruses
ACE2: Angiotensin-converting enzyme 2
RBD: Receptor-binding domain
Mpro: Main protease
MOE: Molecular Operating Environment
